# The cosmological model of eternal inflation and the transition from chance to biological evolution in the history of life

**DOI:** 10.1186/1745-6150-2-15

**Published:** 2007-05-31

**Authors:** Eugene V Koonin

**Affiliations:** 1 National Center for Biotechnology Information, National Library of Medicine, National Institutes of Health, Bethesda, MD 20894, USA

## Abstract

**Background:**

Recent developments in cosmology radically change the conception of the universe as well as the very notions of "probable" and "possible". The model of eternal inflation implies that all macroscopic histories permitted by laws of physics are repeated an infinite number of times in the infinite multiverse. In contrast to the traditional cosmological models of a single, finite universe, this worldview provides for the origin of an infinite number of complex systems by chance, even as the probability of complexity emerging in any given region of the multiverse is extremely low. This change in perspective has profound implications for the history of any phenomenon, and life on earth cannot be an exception.

**Hypothesis:**

Origin of life is a chicken and egg problem: for biological evolution that is governed, primarily, by natural selection, to take off, efficient systems for replication and translation are required, but even barebones cores of these systems appear to be products of extensive selection. The currently favored (partial) solution is an RNA world without proteins in which replication is catalyzed by ribozymes and which serves as the cradle for the translation system. However, the RNA world faces its own hard problems as ribozyme-catalyzed RNA replication remains a hypothesis and the selective pressures behind the origin of translation remain mysterious. Eternal inflation offers a viable alternative that is untenable in a finite universe, i.e., that a coupled system of translation and replication emerged by chance, and became the breakthrough stage from which biological evolution, centered around Darwinian selection, took off. A corollary of this hypothesis is that an RNA world, as a diverse population of replicating RNA molecules, might have never existed. In this model, the stage for Darwinian selection is set by anthropic selection of complex systems that rarely but inevitably emerge by chance in the infinite universe (multiverse).

**Conclusion:**

The plausibility of different models for the origin of life on earth directly depends on the adopted cosmological scenario. In an infinite universe (multiverse), emergence of highly complex systems by chance is inevitable. Therefore, under this cosmology, an entity as complex as a coupled translation-replication system should be considered a viable breakthrough stage for the onset of biological evolution.

**Reviewers:**

This article was reviewed by Eric Bapteste, David Krakauer, Sergei Maslov, and Itai Yanai.

## Open peer review

This article was reviewed by Eric Bapteste, David Krakauer, Sergei Maslov, and Itai Yanai.

### Evolution of the cosmos: eternal inflation, "many worlds in one", and anthropic selection

The "many worlds in one" (hereinafter MWO) model makes the startling prediction that all macroscopic, "coarse-grain" histories of events that are not forbidden by conservation laws of physics have realized (or will realize) somewhere in the infinite universe, and not just once but an infinite number of times [1,2]. There is, e.g., an infinite number of (macroscopically) exact copies of the earth with everything that exists on it, although the probability that a given observable region of the universe (hereinafter *O*-region) carries one of such copies is vanishingly tiny. This picture seems counterintuitive in the extreme but it is a direct consequence of eternal inflation, the dominant model of the evolution of the universe in modern cosmology [3-5].

Inflation is the period of the exponentially fast initial expansion of a universe [6]. In the most plausible, self-consistent inflationary models, inflation is eternal, with an infinite number of island (pocket) universes (hereinafter, simply, universes) emerging through the decay of small regions of the primordial "sea" of false (high energy) vacuum and comprising the infinite multiverse. To observers within each universe, it appears self-contained and infinite, and containing an infinite number of *O*-regions. For such observers (like us), their universe is expanding from a singularity (Big Bang) which corresponds to the end of inflation in the given part of the multiverse. Inflation is in excellent agreement with several crucial results of observational cosmology – above all, the flatness of space in our *O*-region, the overall uniformity of the cosmic microwave background radiation, and its local non-homogeneities [7]. Furthermore, the "populated landscape" version of string theory independently yields a very similar model of the multiverse [8-11]. Thus, although the model of eternal inflation cannot be considered proved, this is the strongly preferred current scenario of the cosmic evolution.

Garriga and Vilenkin showed that, in a finite time, the content of each *O*-region can assume only a finite number of states and, accordingly, any *O*-region has a finite, even if unimaginably vast (on the order of 10^10^150^), number of unique macroscopic, coarse-grain histories [1]. Effectively, the finiteness of the number of coarse-grain histories appears to be a straightforward corollary of quantum uncertainty [2]. The same conclusion is independently reached through a completely different approach, namely, the so-called holographic bound on the amount of entropy that can be contained in any finite region of the universe [1,11,12]. Combined, eternal inflation, the finiteness of the number of unique coarse-grain histories, and the inevitable quantum randomness at the Big Bing (the beginning of time for each universe) lead to the straightforward and striking conclusion that each history permitted by conservation laws of physics is repeated an infinite number of times in the multiverse and, actually, in each of the infinite number of infinite (island) universes [2,11].

The MWO model is tightly linked to the anthropic principle (anthropic selection), a controversial but increasingly popular concept among cosmologists. According to the anthropic principle, the only "reason" our *O*-region has its specific parameters is that, otherwise, there would be no observers to peer into the universe [13-15]. Of course, it should be emphasized that I only discuss here what is often called "weak" anthropic principle and is the only acceptable scientific rendering of this concept. The so-called "strong" anthropic principle is the teleological notion that our (human) existence is, in some mysterious sense, the "goal" of the evolution of the universe; as such, this idea does not belong in the scientific domain. It appears that the anthropic principle can be realistically defined only in the context of a vast (or infinite) multiverse [10]. In particular, in the MWO model, anthropic selection has a straightforward interpretation: the parameters of our *O*-region are selected among the vast number of parameter sets existing in the multiverse (in an infinite number of copies each) by virtue of being conducive to the emergence and sustenance of complex life forms.

Compared to older cosmological concepts that considered a finite universe, the MWO model changes the very notions of "possible", "likely", and "random" with respect to any historical scenario (see Table 1). Simply put, the probability of the realization of any scenario permitted by the conservation laws in an infinite universe (and, of course, in the multiverse) is, exactly, one. Conversely, the probability that a given scenario is realized in the given *O*-region is equal to the frequency of that scenario in the universe. From a slightly different perspective, the usual adage about the second law of thermodynamics being true in the statistical sense takes a literal meaning in an infinite universe: any violation of this law that is permitted by other conservation laws will happen – and on an infinite number of occasions. Thus, spontaneous emergence of complex systems that would have to be considered virtually impossible in a finite universe becomes not only possible but inevitable under MWO, even though the prior probabilities of the vast majority of histories to occur in a given *O*-region are vanishingly small. This new power of chance, buttressed by anthropic selection, is bound to have profound consequences for our understanding of any phenomenon in the universe, and life on earth cannot be an exception.

**Table 1:** Some central new definitions and reinterpretation of familiar definitions in the MWO model

**Term(s)**	**Definition**
**Inflation**	Exponential expansion of the multiverse driven by the repulsive gravity of the false (high energy) vacuum; inflation is likely to be **eternal**, i.e., once started, it will never end.
**Multiverse (megaverse, master universe)**	The entire fabric of reality that consists of eternally inflating false vacuum with an infinite number of decaying small decaying regions giving rise to universes.
**Universe (island universe, pocket universe, bubble universe)**	Part of the multiverse that expands from a Big Bang event resulting from a decay of a region of false vacuum into low energy (true) vacuum. A universe is infinite from the point of view of an internal observer but finite to an imaginary external observer.
**Observable (*O*) region**	A finite region within a universe that can be observed from any given point, i.e., the interior of the past light cone of the given point; our *O*-region contains ~10^20 ^stars.
**Big Bang**	In the traditional 20^th ^century cosmology, expansion of the universe from a singularity; the nature of the "bang" has never been elucidated. In the eternal inflation cosmology, Big Bang corresponds to the end of inflation in the given region of the multiverse as a result of false vacuum decay and the formation of a universe in the form of an expanding bubble of low-energy (true) vacuum.
**Macroscopic (coarse-grained) history**	Any combination of physical events permitted by the laws of physics, characterized to the limit of quantum uncertainty and occurring in an *O*-region within a finite time; it has been shown that the number of all possible macroscopic histories is finite, although vast. Hence even within a single universe, each history is repeated an infinite number of times.
**Probability/chance/randomness**	Textbooks define probability as the limit to which frequency of a specific outcome tends when the number of trials tends to infinity. In an infinite universe (and, obviously, in the multiverse) with a finite number of histories, the infinite number of trials is realized, hence probability equals frequency. The probability of any permissible history including origin of life, then, is *P = 1*. However, the probability *p *of observing any particular history in a given O-region lies in the interval between 0 and 1 as in the textbook definition of probability and can be extremely small for a vast number of histories including the origin of life. Thus, the notions of chance and randomness apply only to finite regions of a universe, whereas in an infinite universe as a whole, the realization of all permitted histories is a necessity.
**Anthropic principle/anthropic selection/anthropic reasoning**	The notion that the history of our world (our *O*-region, our galaxy, our solar system etc) prior to the onset of biological evolution does not depend on any special "mechanism" but was, simply, "selected" from the finite ensemble of all histories that are guaranteed to realize in an infinite universe, by virtue of being conducive to the emergence of complex life. Anthropic selection is an epistemological not an ontological principle, and should not be misconstrued for any kind of active process. This is a formulation of the "weak" anthropic principle adopted for the context of this paper. The "strong" anthropic principle is the notion that the emergence of consciousness, somehow, is a goal of the cosmic history. This is a teleological, non-scientific concept.

### The central problem: the emergence of biological evolution, the inherent paradoxes of the origin of replication and translation systems, and the limitations of the RNA world

The origin(s) of replication and translation (hereinafter OORT) is qualitatively different from other problems in evolutionary biology and might be viewed as the hardest problem in all of biology. As soon as sufficiently fast and accurate genome replication emerges, ***biological evolution ***takes off. I use this general term to include Darwinian natural selection[16] along with other major evolutionary mechanisms, such as fixation of neutral mutations that provide material for subsequent adaptation [17], exaptation of "spandrels" (features that originally emerge as evolutionary by-products but are subsequently utilized for new functions) [18], and duplication of genome regions followed by mutational and functional diversification [19]. All these processes that, together, comprise biological evolution become possible and, actually, inevitable once and only once efficient replication of the genetic material is established.

The crucial question, then, is how was the minimal complexity attained that is required to achieve the threshold replication fidelity. In even the simplest modern systems, such as RNA viruses with the replication fidelity of only ~10^-3^, replication is catalyzed by a complex protein replicase; even disregarding accessory subunits present in most replicases, the main catalytic subunit is a protein that consists of at least 300 amino acids [20]. The replicase, of course, is produced by translation of the respective mRNA which is mediated by a tremendously complex molecular machinery. Hence the ***first paradox of OORT***: to attain the minimal complexity required for a biological system to start on the path of biological evolution, a system of a far greater complexity, i.e., a highly evolved one, appears to be required. How such a system could evolve, is a puzzle that defeats conventional evolutionary thinking.

The commonly considered solution is the RNA world scenario, i.e., the notion that replication evolved before translation such that the earliest stage of life's evolution was a versatile community of replicating RNA molecules [21-23]. A central element of the RNA world is a replicase consisting of RNA. The RNA world concept is supported by the experimental discovery of diverse catalytic activities of ribozymes (catalytic RNAs) [24-27]. However, all the advances of ribozymology notwithstanding, the prospects of a *bona fide *ribozyme replicase remain dim as the ribozymes designed for that purposes are capable, at best, of the addition of ~10 nucleotides to a oligonucleotide primer, at a very slow rate and with fidelity at least an order magnitude below that required for the replication of relatively long RNA molecules [28,29]. As recently noticed by one of the leading RNA world explorers, "Despite valiant efforts,...it appears unlikely that this particular polymerase enzyme will ever be evolved to the point that it can copy RNA molecules as long as itself (~200 nucleotides)" [30]. Of course, it remains possible – and this is, indeed, the belief in the RNA world community – that other ribozymes are eventually evolved to that level; however, the evidence is lacking.

The ***second paradox of OORT ***pertains to the origin of the translation system from within the RNA world via a Darwinian evolutionary process: until the translation system produces functional proteins, there is no obvious selective advantage to the evolution of any parts of this elaborate (even in its most primitive form) molecular machine. Conceptually, this paradox is closely related to the general problem of the evolution of complex systems that was first recognized by Darwin in his famous discussion of the evolution of the eye [16]. The solution sketched by Darwin centered around the evolutionary refinement of a primitive version of the function of the complex organ; subsequently, the importance of the exaptation route for the evolution of complex systems has been realized [18]. However, origin of translation resists both lines of reasoning. Primitive translation in a protein-free system is conceivable as an intermediate stage of evolution (see below) but this does not resolve the paradox because, even for that form of translation to function, the core components must have been in place already. Speculative scenarios have been developed on the basis of the idea that even short peptides could provide selective advantage to an evolving system in the RNA world by stabilizing RNA molecules, affecting their conformations or enhancing their catalytic activities [31-33] (see Ref. [34] for an attempt of a synthesis on this direction in the study of translation origins). These ideas are compatible with observed effects of peptides on ribozyme activity [35] but none of the scenarios is complete or supported by any specific evidence, and all include reactions without precedent in modern biological or model systems.

All this is not to suggest that OORT is a problem of "irreducible complexity" and that the systems of replication and translation could not emerge by means of biological evolution. It remains possible that a compelling evolutionary scenario is eventually developed and, perhaps, validated experimentally. However, it is clear that OORT is not just the hardest problem in all of evolutionary biology but one that is qualitatively distinct from the rest. For all other problems, the basis of biological evolution, genome replication, is in place but, in the case of OORT, the emergence of this mechanism itself is the explanandum. Thus, it is of interest to consider radically different scenarios for OORT.

### The transition from anthropic selection to biological evolution in the history of life and the no-RNA-World scenario

**Figure 1:**
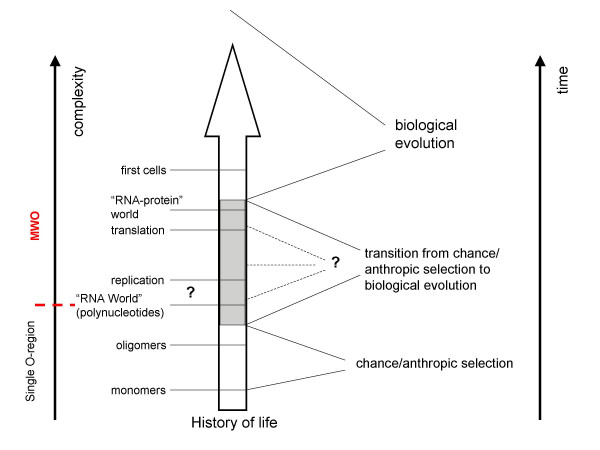
The transition from chance/anthropic selection to biological evolution in the history of life. The grey area and dotted lines illustrate the uncertainty in the identification of the threshold of biological evolution, i.e., the level of complexity at which the transition occurred. The broken red line denotes the boundary between the levels of complexity that, in principle, might be attainable in a finite universe consisting of a single *O*-region from the higher levels of complexity the spontaneous emergence of which would require an infinite model such as the MWO (see Appendix).

The history of life includes a crucial transition from ***chance to biological evolution ***(Fig. 1). Biological evolution cannot take off before there are polymers (most likely, RNA molecules) and means for their sustainable replication. Thus, the synthesis of nucleotides and (at least) moderate-sized polynucleotides could not have evolved biologically and must have emerged abiogenically, i.e., effectively, by chance abetted by chemical selection, e.g., preferential survival of stable RNA species. At the other end of the spectrum, there can be no reasonable doubt that the first cells were brought about by biological evolution. Somewhere in between is the transition, the threshold of biological evolution. Most often, since the advent of the RNA World concept, this threshold is (implicitly) linked to the emergence of replicating RNA molecules. Translation is thought to have evolved later via an unspecified or, at best, invented *ad hoc *selective process. As discussed in the preceding section, both the ribozyme-catalyzed replication and, especially, evolution of translation in the RNA world face formidable difficulties. The MWO model dramatically expands the interval on the axis of organizational complexity where the threshold can belong by making emergence of complexity attainable by chance (Fig. 1). In this framework, the possibility that the breakthrough stage for the onset of biological evolution was a high-complexity state, i.e., that the core of the coupled system of translation-replication emerged by chance, cannot be dismissed, however unlikely (i.e., extremely rare in the multiverse).

The MWO model not only permits but guarantees that, somewhere in the infinite multiverse – moreover, in every single infinite universe, – such a system would emerge. The pertinent question is whether or not this is the most likely breakthrough stage the appearance of which on earth would be explained by chance and anthropic selection. I suggest that such a possibility should be taken seriously, given the paradoxes of OORT. A central corollary to this hypothesis is that the RNA World, as it is currently pictured, i.e., a vast community of replicating RNA molecules possessing a variety of catalytic activities but no translation system and no genetically encoded proteins, might have never existed. Of course, as discussed below, this does not at all rule out the special importance of ribozymes in early biology, in particular, in the primordial translation system.

The modern translation apparatus shows clear signs of evolution by duplication and diversification in the essential, ubiquitous components, allowing one to glean some features of the putative breakthrough system. Analysis of the duplications of key proteins involved in translation suggests that the breakthrough system was an RNA-based machine, to a much greater extent than the modern translation system. Specifically, the aminoacyl-tRNA synthetases (aaRS) comprise two unrelated classes each of which evolved via a series of duplications[36,37]. Moreover, both classes of paralogous aaRS are relatively late elaborations within large classes of nucleotidases [38-40], strongly suggesting that the breakthrough system activated amino acids via an RNA-only mechanism. The same pertains to the translation factors that are relatively late products of evolution within the GTPase class of the P-loop NTPases; thus, the breakthrough system would not employ protein translation factors [41]. The phenomenon of mimicking of tRNA structures by some of the translation factors [42-44] further supports the notion that the ancestral translation system was RNA-centered. The experimentally demonstrated activities of ribozymes include, among others, those that are involved in the main chemical steps of translation, such as amino acid activation, RNA aminoacylation, and peptidyl transfer [45-48]. Self-aminoacylation of ribozymes selected for this activity is rapid and highly accurate, remarkably, even more so than the same reaction catalyzed by the cognate aaRS [49]. Perhaps, most importantly, the large subunit rRNA itself is a ribozyme that catalyzes the peptidyl transferase reaction [50,51]. Thus, an RNA-only translation system, although so far not demonstrated experimentally [52], appears to be a realistic possibility.

A notable and enigmatic feature of the modern translation machinery is the common structure and the presence of conserved sequence elements in the tRNAs of all specificities which suggests that all the tRNAs are ancient paralogs[53]. Thus, the current set of tRNAs, obviously, is a product of biological evolution. The breakthrough system, conceivably, would utilize adaptors that were simpler than tRNAs, with the latter taking over already at the biological evolution stage. These primordial adaptors would have to possess the crucial capacity that, in the modern translation system, belongs to aaRS, i.e., combining amino acids with the cognate anticodons [34].

**Figure 2:**
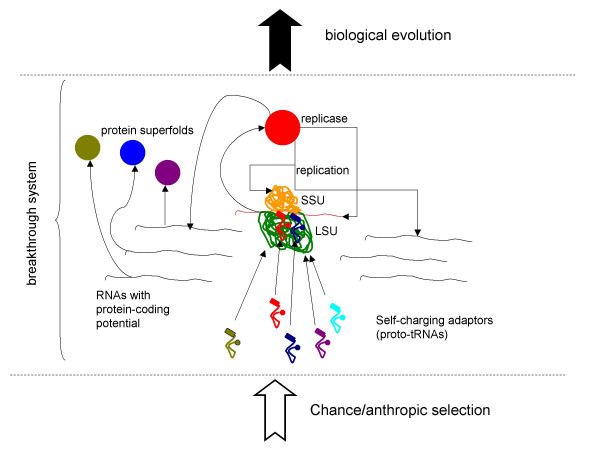
The upper bound for the putative breakthrough system: a primitive, RNA-based coupled system of replication-translation. LSU, large ribosomal subunit; SSU, small ribosomal subunit.

Under the present model, the core elements of the translation system, namely, a RNA-only ribosome and the specific adaptors for, at least, a subset of the 20 modern protein amino acids emerged by chance and were anthropically selected (Fig. 2). The breakthrough system was a primitive, RNA-based translation machine that was capable of translating exogenous RNAs such that functional proteins, including a replicase, could be generated. The presence of a diversity of randomly synthesized RNAs, including one that encoded a protein with a replicase activity (however low, initially), on the early earth would be another anthropically selected feature. For such an ensemble of RNA molecules to exist, a natural "reactor" is required in which polynucleotides are produced at an adequate rate and chemical selection occurs such that stable molecules survive longer. Networks of inorganic compartments existing at hydrothermal vents might be plausible candidates for this role [54,55]. Interestingly, a recent study that combined simulation and experiment has shown that even a low rate of production of mononucleotides would lead to their significant concentration in the peripheral compartments of such networks, and should polynucleotide be formed, they could reach very high concentrations [56]. Thus, the existence of "RNA-making reactors" under prebiotic conditions could be quite realistic [57].

Under these conditions, the emergence of RNA-based translation machinery would lead to the production of the replicase, and, with the ensuing RNA replication, the fundamental transition from anthropic to biological selection would occur (Fig. 2). In principle, the start of biological evolution is imaginable with the replicase initially being the only active protein. However, given the requirement for a RNA-producing "reactor", it seems an attractive possibility that, upon the advent of translation, other random RNA sequences gave rise to prototypes of the other major protein folds, yielding several protein activities (e.g., RNA-binding proteins or primitive enzymes facilitating nucleotide synthesis) and so conferring the minimal required robustness to the emerging biological system. The emergence of these folds would comprise the "Big Bang" of the protein universe [39,58].

The modern, universal genetic code is much more robust than expected by chance with respect to mutational and, probably, also translational errors: it has been estimated that the probability to obtain a code with the same or greater robustness than the actual one is <10^-6 ^[59-61]. This robustness is manifest in the non-randomness of the code structure such that amino acids with similar properties are, typically, encoded by codons that differ in a single position (e.g., all codons with a U in the second position encode hydrophobic amino acids) [62]. This is, typically, considered to be a result of evolutionary optimization of the code [60]. However, the MWO model suggests an alternative view under which the basic structure of the code results from anthropic selection inasmuch as only codes with a certain minimal level of robustness would allow the appearance of a functional replicase in the breakthrough system. Of course, this scenario for the emergence of the code does not preclude subsequent adjustments via biological evolution.

The proposal outlined above eliminates the paradoxes of OORT by postulating that replication and translation, in their most basic forms, have not evolved biologically but rather were brought about by chance abetted with anthropic selection. The MWO model seems to render this a viable, however counterintuitive, possibility.

### Objections, implications, and falsification

The present proposal, the appearance, via anthropic selection alone, of a RNA-protein system sufficiently complex to couple translation with replication such that biological evolution could take off, might seem quite outrageous. However, there are several mitigating considerations. First, the postulated chance origin of the replication-translation system does not require any mysterious processes. On the contrary, the only reactions involved are regular ones, such as polymerization of nucleotides and amino acids, nucleotide phosphorylation/dephosphorylation etc, and the only interactions required are those that are common in chemistry and biochemistry. Interestingly, the elementary reactions required for translation (amino acid activation, RNA aminoacylation, and transpeptidation) are relatively easily modeled with ribozymes (see above), in a marked contrast to RNA replication. Second, any conceivable scenario of life's evolution necessarily requires combinations of highly unlikely conditions and events prior to the onset of biological evolution, including the abiogenic synthesis of fairly complex and not particularly stable organic molecules, such as nucleotides, the concentration of these molecules within appropriate compartments, and their polymerization yielding polynucleotides of sufficient size and diversity. Thus, anthropic selection appears to be an inevitable aspect of life's evolution (Fig. 1).

Here I invoke the MWO model to argue that the range of complexity that is open to anthropic selection could be much greater than previously suspected such that a primitive coupled replication-translation system might have emerged without biological selection (Fig. 1). This scenario seems to eliminate the paradoxes of OORT. The origin of a complex system capable of performing a biological function by chance might appear nonsensical. I believe, however, that this is, primarily, a semantic trap. Prior to the onset of biological evolution, there could be no function, just complexity, and the emergence of any level of complexity is guaranteed by the MWO model.

**Figure 3:**
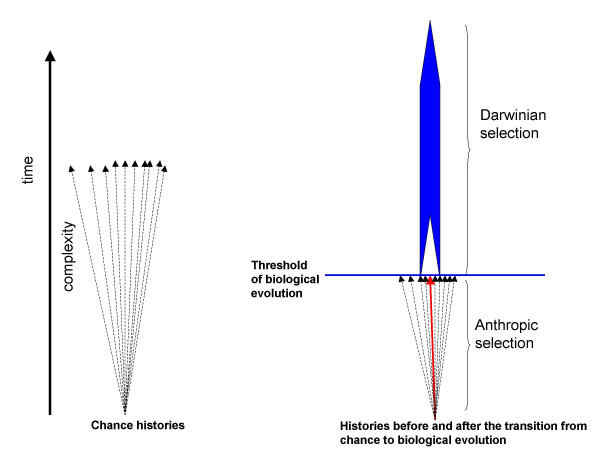
The narrowing of the range of possible histories and the increased likelihood of the emergence of high complexity brought about by the transition from chance to biological evolution. The thick read arrow shows the history that leads to the breakthrough system.

A crucial aspect of the framework developed here is brought about by a disturbing (almost nightmarish) but inevitable question: in the infinitely redundant world of MWO, why is biological evolution, and in particular, Darwinian selection relevant at all? Is it not possible for any, even the highest degree of complexity to emerge by chance? The answer is "yes" but the question misses the point. Under the MWO model, emergence of an infinite number of complex biotas by chance is inevitable but these would be vastly less common than those that evolved by the scenario that includes the switch from chance/anthropic selection to biological evolution. The onset of biological evolution canalizes the historical process by reducing the number of available trajectories to the relatively few robust ones that are compatible with the Darwinian mode of evolution of complex systems (Fig. 3). This leads to a much greater rate of change than achievable by chance such that, as soon as there is an opportunity for biological evolution to take off, anthropic selection is relegated to a secondary role in the history of life. Of course, "secondary" does not mean unimportant; contingency and randomness are crucial, especially, at transitional stages of evolution (e.g., [63,64] but the basic framework is Darwinian. Thus, in any reconstruction of the origin of life, the threshold should be mapped to the lowest possible point, i.e., to the minimally complex system capable of biological evolution.

The strong form of the present hypothesis, i.e., the notion that the breakthrough stage in the history of life was a primitive coupled replication-translation system (Fig. 2), is falsifiable. Such a system should be construed as the upper bound of complexity for the breakthrough stage (Fig. 1). As soon as the possibility of biological evolution at a lower level of complexity, e.g., in the RNA world, is demonstrated and the route from the RNA world to the translation system is established, either experimentally or, at least, in a compelling model, the origin of a complex system with coupled replication and translation by chance/anthropic selection will be, effectively, ruled out. A demonstration that life independently emerged on several planets in our *O*-region will have the same effect. In the Appendix, I provide a rough, toy calculation of the upper bound of the probability of the emergence of a coupled replication-translation system in an O-region – this probability is, indeed, vanishingly small. The converse prediction is that any life forms that might be discovered on Mars or, perhaps, Europa during future planetary explorations will have a common origin with life on earth.

Any of the above falsifications will refute the model shown in Fig. 2 but will not make the MWO worldview irrelevant for our understanding of the origin of life. Indeed, any such discovery (tremendously important in itself) will simply lower the threshold of biological evolution on the scale in Figure 1. This point can be illustrated by deliberately naïve, toy calculations of the upper bound of the probability of the emergence of different versions of the breakthrough system by chance. In the Appendix, I present such calculations for two versions, the RNA World with a ribozyme replicase, and the coupled translation-replication system. Under the assumptions of this toy model (idealized to the extreme in that an unrealistically high rate of abiogenic RNA production is assumed), the emergence, by chance, of a ribozyme replicase in a finite universe consisting of a single *O*-region like ours, in principle, could be considered. However, any significant increase in complexity would call for a different cosmological model. In particular, the emergence of a coupled replication-translation system is unlikely to the extent of being, effectively, impossible. For such a complex system to be a viable candidate for the breakthrough stage, an infinite multiiverse, such as the one depicted by MWO or, in the very least, a universe with a vast number of *O*-regions, is, indeed, a must.

Of course, the most straightforward and powerful falsification would be to disprove the MWO itself. Here, however, an important disclaimer is due. It is not, actually, crucial for the validity of the conceptual framework presented here that MWO is correct in all its details. Only two rather generic assumptions are essential: i) a spatially infinite universe such as any (island) universe in MWO; the multiverse, while integral to eternal inflation, is not actually required for my argument, ii) the finiteness of the number of macroscopic histories in any finite region of spacetime. The strong form of the hypothesis presented here will not be falsified if some specific details of the MWO turn out to be wrong but only if one of these general assumptions fails.

## Conclusion

Despite considerable experimental and theoretical effort, no compelling scenarios currently exist for the origin of replication and translation, the key processes that together comprise the core of biological systems and the apparent pre-requisite of biological evolution. The RNA World concept might offer the best chance for the resolution of this conundrum but so far cannot adequately account for the emergence of an efficient RNA replicase or the translation system.

The MWO version of the cosmological model of eternal inflation could suggest a way out of this conundrum because, in an infinite multiverse with a finite number of distinct macroscopic histories (each repeated an infinite number of times), emergence of even highly complex systems by chance is not just possible but inevitable. This dramatically expands the interval on the scale of organizational complexity where the transition from anthropic selection to biological evolution might belong. Specifically, it becomes conceivable that the minimal requirement (the breakthrough stage) for the onset of biological evolution is a primitive coupled replication-translation system that emerged by chance. That this extremely rare event occurred on earth and gave rise to life as we know it is explained by anthropic selection alone. Under this model, a full-fledged RNA world, with a diverse population of replicating RNA molecules but without translation, was not a stage in the origin of life on earth. However, this does not defy the central role of RNA in the emergence of biological evolution and early evolution of life. Indeed, the model includes a complex ensemble of non-replicating RNA molecules as the product of anthropic selection that enabled the onset of biological evolution.

Connections between biological evolution and cosmological models have been proposed previously as analogies. Shakhnovich and coworkers developed a simple mathematical model of an "expanding" protein universe that they aptly likened to the Big Bang model of the evolution of the physical universe [58]. From the cosmological side, Smolin proposed the model of cosmic selection that extended the Darwinian principles to the evolution of the universe[65,66]. By contrast, here I propose a direct link between specific models of evolution of the physical and biological universes, with the latter being contingent on the validity of the former (MWO) as illustrated by simple calculations. Importantly, in this context, the validity of MWO is to be understood in a rather generic sense. For the present concept to hold, the only essential assumptions are that the universe is infinite [e.g., any (island) universe under MWO; the multiverse, per se, is not a must] and that the number of macroscopic histories in any finite region of spacetime is finite.

A final comment on "irreducible complexity" and "intelligent design". By showing that highly complex systems, actually, can emerge by chance and, moreover, are inevitable, if extremely rare, in the universe, the present model sidesteps the issue of irreducibility and leaves no room whatsoever for any form of intelligent design.

## Reviewers' comments

### Reviewer 1: Eric Bapteste (Université Pierre et Marie Curie)

I might be strange but I often like to read Eugene Koonin's papers. This one, about the origin of replication and translation mechanisms within a particular cosmological framework, is vastly metaphysical. Biologists might appreciate that it addresses the limits of application of classical evolutionary thinking to early life stages in a pretty original way. Developing rigorous metaphysical arguments – especially when dealing with cosmological scenarios- is however very tedious. It probably requires a solid philosophical background to be done convincingly, and most likely the collaboration of both evolutionists and philosophers to write such an ambitious manuscript. A collaboration of that kind would help clarifying the meaning of many of the terms used and help presenting what specialists of the field consider to be the important notions and issues in cosmology, possibly coupled with a fair introduction for biologists about the alternative cosmological main models.

In my short review of this paper, I will assume that, although there is no doubt Eugene Koonin is a very bright evolutionist and a powerful thinker, he can not be considered as an expert on cosmology. For this reason (maybe), I feel that, if there are positive aspects in this manuscript, there are also important sources of concerns in it.

**Author's response**: *There are a great many issues touched upon in this not so short review, and replying in full would, effectively, take another paper. Furthermore, to write such a paper properly, one might indeed need to be (or closely collaborate with) a professional philosopher. I will therefore restrict myself to a few brief comments on specific points, and a closing statement on the relationships between physics and metaphysics, and between the Anthropic Principle and Intelligent Design; additional relevant discussion can be found in my response to Krakauer below*.

As a researcher, I welcome the multiplication of alternative scenarios (metaphysical – when coherent- or scientific) to address scientific questions. This pluralistic approach is probably the best way to test our favourite scientific theories by challenging them, in any case to keep them alive and debated.

Eugene Koonin's present appeal to cosmology in order to address a complex evolutionary issue tells me that, as a scientist, **he **feels that there is currently a problem with the scientific theory aiming to explain the origin of replication and translation. In other words, his approach says something both about him and about the possible limits of our discipline on that topic. In this review, I will simply try to rephrase what serious problem Koonin has identified according to me, and I will argue that I am afraid his answer to this problem might open too broad an avenue to the supporters of intelligent design, as it is currently formulated, and thus does not satisfy me as such as an alternative to the theory to the RNA world.

### Koonin's problem and solution

Koonin's problem (the central problem of biology, he claims! (cf.p.6)) is that traditional evolutionary models can not help conceiving the emergence of replication and translation systems: for him, they would be too complex to have evolved independently of a primordial replication-translation system by natural selection acting on ribozymes alone. To solve this issue, Koonin proposes that a coupled complex system of translation and replication emerged instead by chance and by anthropic selection. After what he assumes that Darwinian selection took off and that the traditional evolutionary thinking can be applied safely to analyse the rest of the biological evolution.

To overcome the fact that the likelihood of the emergence of a complex system by chance alone is low, Koonin's multiplies the universes where such a phenomenon could be observed. Although some event *e *is deemed to be rare in one universe, in a multiverse framework, there must be one universe where such *e *happened. In his own terms: "spontaneous emergence of complex systems that would have to be considered virtually impossible in a finite universe becomes not only possible but inevitable under MWO, even though the prior probabilities of the vast majority of histories to occur in a given O-region are vanishingly small" (p. 6).

This position raises itself many concerns (biological and philosophical):

- Is it really true that traditional natural selection could not explain the emergence of the replication and translation systems?

- In any case, why should we assume that the emergence of translation and replication had to be coupled in the first place? Would not it simplify the "central problem" (i.e. make one of these questions amenable to classical evolutionary thinking) if both of these issues were uncoupled? For instance, assuming replication evolved first, could not it be possible that translation evolved progressively under Darwinian selection?

- Is a transition from anthropic selection to Darwinian selection possible and likely?

- Is it possible to assume such a transition without taking the high risk to reintroduce teleology everywhere in the evolutionary field?

Deeply, I agree with Koonin that explaining the origin of replication cycles in general challenges any sort of thinking based on natural selection and goes beyond the classical evolutionary theory, in the sense that Darwinian evolution needs replication to happen and evolutionists need replication cycles (and descent) to reason. Explaining the origin/cause of the phenomenon of replication is thus a big problem. Yet, I disagree with Koonin that natural selection and cycles of replication have to happen late in the cosmological history. To me it (only) takes a population of imperfect replicators of whatever nature for the natural selection to act, and some models on early life suggest how molecules, crystals of some sort could replicate on different inorganic substrates. Once replication is in place, and from a traditional selectionist perspective, it does not seem impossible that emerges a biological system of minimal complexity from which the process of biological replication itself could evolve under natural selection. In other words, I would locate the so-called "central" problem one step earlier than Koonin does in his manuscript: if we (evolutionists) were asked to explain replication in general, we might get in trouble, and we might be seriously tempted by metaphysical arguments.

Koonin bravely tries to tackle such a deep conceptual issue, using metaphysics where, according to him, science does not seem to work, but I am afraid his present (and arguable) solution, although fairly underlining one of the limits of traditional evolutionary thinking, could open a huge door to the tenants of intelligent design.

### My general issues with the manuscript

#### Philosophy or biology or both?

##### At various locations, precisions are needed to allow a real understanding of the first (cosmological) section of the paper

Maybe because my background in both biology and philosophy is limited (I have a phD in both), I have a general issue with several parts of the manuscript, which should be either more rigorously developed or should be significantly shortened. At the very least, I believe that to be truly understood the author must propose -very early in his manuscript- clear definitions (in a box, or in the main text) of the following terms:

Cosmology, probable, possible, multiverse, anthropic selection, Darwinian selection.

**Author's response**: *I took up this suggestion and included some of such key definitions in a Box. There was no need to define Darwinian selection, in my opinion, but the less common key notions, and those common ones that are reinterpreted here are defined*.

He can not just assume that every reader shares his conceptions of the meaning of these different notions. He should also clarify what in biology are "macroscopic histories" by giving biological examples of it. In general, he should make clear when he is using a word in its philosophical sense or in its more common meaning. For instance, I am not sure what Koonin means by "repeated" in his sentence: "each history permitted by conservation laws of physics is repeated an infinite number of times in the multiverse" (p. 5). Does he mean each history is duplicated, exactly identical, an infinite number of times (i.e. the very same organism reoccurred in the very same context endlessly)? Depending on what the source informing his opinion was (philosophical or not), there might be a possible confusion. Indeed for the process-philosophers (who come to my mind and who have tried to develop a cosmology), repetition never means repetition of the same, but all the time repetition of something different (see Deleuze for instance).

**Author's response**: *Let us be clear: not a single term in this paper is used in any specific philosophical meaning. The cosmological models and concepts discussed here are physics not metaphysics, even as they have important philosophical implications (see my response at the end of this review for a more general discussion)*.

#### First major concern: should we reintroduce teleological processes to explain what can not be explained by natural selection?

Biological systems replicate, yet Koonin tells us we can not know how they happen to do it in the first place and that we need to seek for an answer outside of the realm of traditional evolutionary thinking to address this question. He calls in multiverse and anthropic selection.

I have particular trouble with this second notion.

If Koonin really wants to promote the anthropic principle the way he defines it – ("According to the anthropic principle, the only "reason" our O-region has its specific parameters is that, otherwise, there would be no observers to peer into the universe", p.5)-, he seems to be assuming no less than a major role for teleology to explain life biodiversity (namely here that the evolution of observers to peer into the universe is the root cause of evolution). In general, teleology means that processes are directed by their goal-to-be-achieved, that their real causation is the later consequence the structures developed in the evolutionary process will lead to. It certainly makes sense in a context of replication (like descent with modification and natural selection) that a selected effect (i.e. the possession of a phenotype) can act as a cause on the evolution of future organisms of the same lineage (i.e. if the development of an eye fulfils a function of perception, therefore it can be selected if it increases the fitness of perceptive organisms in the population). However, assuming that teleology is acting before the replication process and natural selection are started is equivalent to assume that there is an *a priori *reason for which there has to be one day, on earth, organisms with eyes, whose goal will be to see. Unfortunately, such a position does not distinguish itself from the conception of the supporters of intelligent design either. ID people could always claim that life evolves for a reason (they could also enrole Darwinian selection as a mean to achieve this higher goal/reason).

Thus, I want to precise that I do not see what's really brought in by invoking such an "anthropic principle" (but trouble). The idea that evolution would be oriented toward consciousness is a recurrent cosmological option indeed but it is definitely not the only one possible, and certainly not mine. A deeper philosophical exploration of cosmological theories would most likely have showed that evolutionists do not have to embrace a strong anthropic principle as if it was an integral part of the cosmological package introduced here (too roughly) by Koonin.

Yet, maybe Koonin himself does not care so much for this anthropic principle, as I suggest below.

**Author's response**: *this is a very, very serious misunderstanding of the anthropic principle. The idea that anything is "oriented toward consciousness" is pernicious nonsense. The revised version of the paper includes a brief debunking of the so-called "strong anthropic principle", in response to this unfortunate misstatement and a similar one made by Krakauer. I have to be frank here: to me, this mistake invalidates much (not all) of the critique in the review. There is no teleology at all involved in my approach in this paper. No teleology. It is the opposite of teleology. More on this in my closing response to Bapteste*.

#### Second concern: assuming Koonin's model, are there strong evidence of the transition from anthropic selection to Darwinian selection?

Koonin thinks that anthropic selection is inevitably replaced by Darwinian selection once a sufficiently efficient mechanism of replication/translation has been put together. Thanks to such a transition, the teleological order of things would thus be replaced by the Darwinian order of things, under the action of natural selection. (In other words, once his problem is solved, Koonin does not need alternative models to evolutionary thinking anymore). Unfortunately, as long as he does not carefully introduce and clarify his anthropic selection principle, I think that he might be unwillingly opening a huge door to ID supporters to enter into the very heart of the evolutionary theory.

What guarantee do we have indeed that Darwinian selection would replace anthropic selection?

What guarantee do we have that some anthropic selection would not persist, having some effects on the living world, although of lesser importance than those of Darwinian selection? What guarantee do we have that it would not be still acting on and then be responsible, here and there, for some lucky natural "miracles"? (i.e. the birth of a pandemic, a spontaneous generation, a benevolent mutation for superior organisms, any kind of oddities (toward consciousness), since after all the anthropic principle does not say if the observers to peer (that peer? peering?) into the universe have to come sooner rather than later, does it?) ?

**Author's response**: *Again, there is absolutely no teleology involved, I could not insist more strongly on this point. Beyond that, however, we certainly cannot have a guarantee that the anthropic principle was not involved in subsequent evolution because it definitely was. Certainly, in the context of the infinite multiverse, there is an enormous number of worlds where eukaryotes have never evolved...or animals etc. The history of life is riddled with contingency, and there is nothing new about this notion. Not that I am so keen to rely on authority but leading scholars of evolution from Jacob to Monod to Gould advocated the importance of contingency with great force. Gould's metaphor of rewinding the tape of evolution – and finding that even major events would not be the same – is, perhaps, most vivid. A prominent philosopher, Dan Dennett, also wrote about this in "Darwin's Dangerous Idea" and even claimed that evolution is governed by deterministic chaos. An unsettling description, perhaps, but essentially true, I believe. Thus, the anthropic principle remains relevant throughout the history of life although, after the breakthrough stage (see *Figs. 1* and *3), *it is, largely, subjugated by the Darwinian process. I included a brief comment to this effect in the revised manuscript*.

Worse: what guarantee do we have that there won't be ID people to claim that, as the very important evolutionary biologist E. Koonin showed, Darwinian selection is a secondary player in cosmology, and is itself a force evolved for a reason, a chosen by product of anthropic selection, in which case everything evolving under Darwinian selection evolves in fact under the eternal drive of the anthropic principle? (As I read it, in this paper Koonin did not prove that anthropic principle and natural selection were not working hands in hands, and that the later was not following the former for a reason of higher order).

For all these questions could be exploited very meanly by ID people, Koonin is in my view very naïve to think that he can call in teleology to start the process of evolution and call it off subsequently: if teleology is called in (a view which I doubt is worth supporting in science and that I feel is dangerous), unless teleology achieves the goal it is needed for, there is no reason why teleology should suddenly stop being relevant in the middle of the way... In fact, a teleological process deemed to be non successful is no more a force, as by definition it will fail to achieve its goal, and then why to invoke it as a force in the first place?)

Does Koonin really want to claim that "the basic structure of the [genetic] code results from anthropic selection in as much as only codes with a certain minimal level of robustness would allow the appearance of a functional replicase in the breakthrough system" (p.13), and take the risk to be badly misinterpreted? Obviously, he is smart enough to foresee this kind of problems and the reason why he claims he does not worry about it (p.18) is perhaps that this kind of "anthropic selection" is not really what he has in mind.

### Third concern: is our world Koonin's brave new world?

Maybe, instead of invoking such a strong anthropic selection, Koonin is arguing along much simpler lines. I suspect he is in fact introducing a model from which teleology could be entirely absent to justify that, if everything with a low probability is possible somewhere, even though it has a low probability in a given universe, providing a high enough number of universes, everything -including the oddest phenomena- becomes necessary somewhere. Then, complex structures for replication have to emerge. By chance, we happened to be in the "right" type of universe where this very emergence took place, says Koonin. It is just maths: the initial paradox can be solved by an axiom: it had to be so or we would not be here to tell. Now, some people (me included) might find this position to overcome the paradoxical origin of replication a little bit dry (and panglossian).

**Author's response**: *So Bapteste, after all, understands what I am trying to say. Is the whole basis of the criticism above the mistaken identification of anthropic principle with its ridiculous strong version? Very regrettable if so. In the revised manuscript, I stress this point in the text and in the Box. Hopefully, this eliminates any cause for confusion. Now, whether or not the concept developed here is dry is, of course, just a matter of taste. However, the characterization of my position as "Panglossian" cries for a comment. I believe that the worldview presented here is, actually, anti-Panglossian. Indeed, in an infinite multiverse with a finite number of histories, there is no chance that we live in the best of all possible worlds. There is an infinite number of worlds that are incomparably better, even those where Elvis is still alive in 2007 as wryly noticed by Garriga and Vilenkin (refs. 1 and 2). Thus, I believe that here I am relegating Dr. Pangloss to irrelevance, continuing along the lines of Gould and Lewontin's famous San Marco paper*.

Especially if this kind of claim is applied again and again to several complex problems we are (and we will be) facing. As Koonin notices, even if it is unlikely, one could indeed propose that the whole life organization of a universe is just the necessary outcome of such a model. That's why in his paper, a central question subsequently becomes to decide up to which point complex life phenomena to be explained on our planet were necessary rather than the result of Darwinian selection (i.e. at which point traditional scientific explanations become valid again). Regarding that issue, Koonin takes a strong position: when the conditions for Darwinian selection are satisfied (namely once biological replication and translation systems have been set up), necessity leaves our world. (Someone with a different "central" problem might thus observe that, when Koonin does not need necessity anymore to play the role of the explanans of his own biological problem, he drops it; yet maybe another "central" problem still challenges traditional evolutionary thinking, should we invoke the necessity and the mutiverse for it one more time?).

If what I described is what Koonin had in mind rather than the anthropic selection, the good news then is that since such necessity is strictly supported by statistics, he can propose that things are the way they are because they had to be so, without needing to invoke observers of the universe and to open the door of evolutionary biology to ID people.

**Author's response**: *I appreciate these points. The wording in the original manuscript was unnecessarily rigid, in an attempt to strongly emphasize the transition. I made modifications to acknowledge the important role of contingency at subsequent stages of evolution as well*.

Yet, scientists could wonder if this principle position (use necessity when the Darwinian model does not apply, else use the Darwinian model) is the only and best null hypothesis to deal with issues regarding early life. They could notably question:

- how much time is needed for everything to happen somewhere in the multiverse (and if this is a realistic time line from our human perspective),

- if this multiverse scenario is significantly more likely than chance events on a single universe,

- if it is that impossible for populations of ribozymes to be self-sustaining and self-replicating?

Koonin tries to address these questions with some toy statistical models, yet I am very skeptical that toy probability calculations can prove decisive in that matter. (For instance, Whitehead's cosmology (a solid philosophical reference but not an easy read) talks a lot about societies and populations, nexus of all kinds of elements, which could have emergent properties, and could offer interesting breakthroughs, the probabilities of which are probably impossible to derive mathematically).

To summarize, it is legitimate that Koonin tells us where he feels comfortable applying traditional evolutionary thinking. It is legitimate that he tries to propose possible explanations to go beyond that limit. Yet, I am not convinced that there is more than intuition in this paper and that he could prove that our world (the explanandum) is the brave new world he, as a scientist, would be happy with.

I am thus interested -and amused- that, in this paper, Koonin ends up multiplying the universes to solve a biological issue, when I remember how much, on other occasions, he enjoys to invoke parsimony (the assumption that one should not multiply beings without necessity). I would be more satisfied however when he would have made his own views on ID and the anthropic principle clearer in a revised version of this manuscript.

**Author's response**: *Considering the main points of Bapteste's comment, I believe I need to summarize my position on three issues: i) physics versus metaphysics, ii) the nature of the anthropic principle, iii) the relation of the present concept to Intelligent Design (ID). Obviously, the first point can be discussed at great length, so I will just say it a nutshell. The concept developed here is a purely scientific one, positioned at the interface of physical cosmology and straightforward evolutionary biology. If some aspects of the paper seem unusual and counterintuitive, that is mainly because such are the key features of the MWO model. Of course, the MWO model has profound philosophical (both metaphysical and epistemological) implications (see refs. 2 and 13 for discussion), and the biological implication developed here has additional ones. There is nothing unique in this: when the fundamental aspects of the structure of the world are concerned, physics and philosophy merge. In the most obvious example, foundational research in quantum physics had been like that for the last 80 years*.

*I have already made several statements on the anthropic principle above, but I think a more definitive brief explanation should be useful. Once again, we deal here only with the weak anthropic principle that has nothing to do with any teleology. Moreover, it is a fairly trivial statement, "pure math" as Bapteste puts it. In an infinite multiverse, where all possible scenarios actually realize, albeit with very different probabilities, we naturally find ourselves in one of those, extremely rare O-regions where the conditions are conducive to the evolution of complex life forms. This is all there is to anthropic selection which it is not any kind of active selection process. This concept is the very opposite of any kind of teleological scenario: there is nothing special about our region of the universe except that it belongs to the relatively small biophylic domain of the parameter space of the multiverse (e.g., refs. 14,15), and even within that domain, a region with such properties is rare (the fact that this region is very special to us because we live here is scientifically irrelevant). Effectively, under this concept, the conditions for the onset of biological evolution emerge by the power of large numbers, without the involvement of any special interactions let alone any directional process. I think this is the ultimate anti-teleogical stance. To conclude the discussion of the anthropic principle, it might be helpful to emphasize, once again, its crucial link to the multiverse model. In a solitary universe, as depicted by the classical Big Bang model, anthropic principle would amount to enormous luck; that, indeed, would be a Panglossian world. In the infinite multiverse, the element of luck is removed, and the emergence of an infinite number of instantiations of life is guaranteed as long as life is compatible with the laws of physics (the one example we are familiar with proves that it is), even if the biophilic regions of a universe are enormously far between. I would like to quote a rather categorical statement of Leonard Susskind (the inventor of the original version of string theory) on the connection between the multiverse (megaverse, in his parlance) and the anthropic principle: "Without the idea of a megaverse of pockets, there is no natural way to formulate a sensible Anthropic Principle" (Ref. 8, p. 300)*.

*The main text of this paper contains a clear statement on Intelligent Deisgn (ID) but, since this is a serious concern for Bapteste (and I agree that ID is an important, even if meta-scientific, issue), I will reiterate and further reinforce my position that directly follows from the reasoning explicated in this paper. The above discussion of the anthropic principle implies an unequivocal sentence for Intelligent Design; let us spell it out. As indicated above, in an infinite multiverse, anthropic selection guarantees the emergence of systems of whatever complexity that are required for the biological evolution to take off (see *Fig. 3). *This scenario is watertight: first, chance/anthropic selection, then biological evolution. There is no gap here where the ID wedge could fit. Properly interpreted, the anthropic principle is a death knell to ID*.

*Obviously, anthropic principle is often misinterpreted as providing support to religious (and otherwise teleological) believes (regrettably, this includes Bapteste's review). On this issue, I cannot resist quoting the recent book by Richard Dawkins: "It is a strange fact, incidentally, that religious apologists love the anthropic principle. For some reason that makes no sense at all, they think it supports their case. Precisely the opposite is true. The anthropic principle, like natural selection, is an alternative to the design hypothesis. It provides a rational, design-free explanation for the fact that we find ourselves in a situation propitious to our existence" *(Dawkins, R. 2006. The God Delusion, Houghton Mifflin, Boston-NewYork, p.136).*Just like Dawkins, I find it difficult to identify the exact reasons behind the confusion (though see further discussion in my response to Krakauer's review). The possibility that the ID crowd interprets this paper as support for their cause is one of Bapteste's main concerns. Will they, actually? No doubt they will! However, the only way to prevent them from doing so is to stop publishing research on any hard problem in evolutionary biology and somehow declare these problems solved. The ID folks do no research themselves, so they apply all their considerable intellectual resources to turn published scientific work upside down and claim support for ID (it happened to several seemingly innocuous papers of mine, to my considerable amusement). I believe evolutionary biologists should not and actually can not worry about this, only about their own papers being correct and coherent*.

## Reviewer 2: David Krakauer (Santa Fe Institute)

As this paper is somewhat unusual, consisting more of a philosophical contribution at this stage than a scientific one, my review will be concordantly, somewhat unusual. I should declare at once that I do not share Professor Koonin's perspective on the early origin of life and the role of what is sometimes called " anthropic" reasoning or the self-selection principle. Anthropic reasoning has proved to be of some utility in string theory where the superabundance of meta-stable low energy vacua (or distinct universes with distinct parameters) realized through inflationary mechanisms, has proved a challenge to those projects seeking to derive our universe from first principles. The weak anthropic principle seeks to determine what we can expect to observe by the conditions neccessary for our presence. The principle first suggested by Carter is a perfectly scientific statement, and somewhat surprisingly has value in allowing us to apply Bayesian inference to cosmological phenomena. Carter was also the first to apply anthropic principles to the origin of life in 1983, where it was argued that many steps in the early evolution of life might have been fairly improbable, an argument pursued at greater length in this contribution. My principal objection throughout this review will be that the anthropic principle is only scientific in so far as it can do some work, in allowing us to calculate different states of order. Without the calculation it remains an interesting metaphysical insight.

**Author's response**: *I do not strongly disagree except that I believe that the demand for "calculation" is unnecessarily narrow. To be scientifically useful, a principle (concept, hypothesis etc) must offer testable predictions, quantitative or qualitative. Both kinds of predictions are given in this manuscript, qualitative ones in the body of the paper, and quantitative ones in the Appendix. Granted, the latter are toy calculations but calculations nevertheless. Thus, by this simple criterion, this work is "physics" not metaphysics, even as metaphysical implications abound*.

The Koonin Hypothesis (KH for short) is clearly stated on p12 of the manuscript, "The core elements of the translation system, namely a RNA -only ribosome and the specific adaptors for, at least, a subset of the 20 modern protein amino acids emerged by chance and were anthropically selected." In other words, one of the core structures upon which all of life depends arose in a series of highly improbable steps. Following the theory of large deviations, replication and translation emerged as an exponentially unlikely event in proportion to its deviation from a suitable equilibrium ground state. This we might call an *"ahistorical" *theory of proto-biology.

**Author's response**: *I have no objection to Krakauer's statement that the quoted phrase formulates KH; let it be so. However, to completely disambiguate the issue, I should reiterate that KH is just a strong, extreme version of a more general framework that, in the same tongue-in-cheek style, I will denote KC (Koonin Concept). The KC can be stated thus: ***biological evolution started when the minimal complexity required for that was reached through processes governed by anthropic selection**. *Further, I think that defining KC/KH as "ahistorical" is somewhat misleading. There is history involved, for sure, just not biological evolution/selection. Speaking of these concepts as "non-selectionist" or "non-adaptationist" would be more accurate*.

The history of science, is by one reading, interpreted as a catalogue of conceptual bottlenecks overcome by synthesizing unlikely concepts. The characteristics of a bottleneck are: an inability to make progress based on contemporary theory and data, a general impetus to search for radically new ideas, and a correlated tendency to default to extra-scientific modes of explanation based on putative forces and miracles. The general theory of relativity, quantum mechanics and natural selection all solved hard problems by combining hitherto unrelated concepts – non-Euclidean geometry and gravity, probability and mechanics, density regulation with environmental selection. Interestingly, all three theories have their non-scientific resonances, "everything is relative", "everything is subjective", and "everything was created" descending from the bottleneck period. The origin of life will require just such juxtapositions and I find that resorting to extreme fluctuation reasoning, such as in the KH, somewhat unsatisfactory

I think that the KH falls short of being a scientific statement as it invokes events with no well defined probability measure, and in addition, eschews identifying mechanisms.

First, with regard to probability spaces, recall that any probability space includes a sample space of events (which we can think of as the power set), a set of outcomes or events (non-empty subsets) the sigma algebras, and a probability measure that assigns a real value in the interval 0 to 1 to the events. This value is analogous to a volume in a state space, and tells us how likely it is that an event takes place. Now not all events in the power set have a Lebesgue measure, in other words, there are those to which we can not assign a volume. This is not as strange as it seems, as the rational numbers have exactly this property. In the KH we are provided with no means of calculating the probability distribution over biophylic universes, just the statement that any universe is possible. The anthropic principle does the work of extracting our universe by *a posteriori *declaring that we are in one. This is logically correct but operationally without value. It has been stated that the whole history (and future) of the world exists in the decimal expansion of pie (assuming that it is random). But the probability that we would find this history is indeterminate. This is what I worry about in the KH, that there is no way of assigning a measure to the replication and translation machinery in the multiverse.

In this paper we have neither any mechanism or constraints for the translation system, nor prior observations on which to base our probability measures. We have only a conjectured sample space, and no systematic mechanism of coarse graining the relevant measures. This leads to a rather strange outcome where we might as well assert that all observed biological order emerged in one step, including the complete evolutionary history of life. This is equally as possible as the emergence of an RNA polymer, and eye or an atom. Notice that these are all equally possible, namely certain in an infinite multi-verse, as there is no conceivable way of deciding whether they are all equally probable without first writing down an appropriate equilibrium theory for the universal ground state, by making use of relevant cosmological data etc. in our local space.

**Author's response**: *A probability measure for any events is readily defined in the MWO multiverse – in fact, the definition of probability is simpler and more natural than it is in a finite world inasmuch as probability and frequency become one and the same (see the new *Table 1* I made per Bapteste's suggestion)*.

Now let's consider mechanisms. Mechanisms represent our most fundamental intuitions about causality; mechanisms posit relationships between two or more observables based on a theory of interactions. Mechanistic theories are always provisional, but their utility can be inferred through their ability to provide useful predictions out of sample. Mechanisms offer an alternative to observation, by interpolating and extrapolating from a finite body of data. Statistical associations are useful but they are limited to a single history of observation. Mechanisms rise above history and are capable of describing an ensemble. Darwin's theory derives its power not from its ability to explain the bill of a finch, the shell of a snail or the leaves of the mimosa plant, but because it can account in part for all of these, and indeed any, adaptive system in nature, on earth or elsewhere.

In this paper we are provided with no constructive algorithm for arriving at a probability measure for translation or any natural mechanism by which it might come about. The reason why the KH stops short of invoking spontaneous creation for all levels or organization, is that it is confident that we have a suitable theory – Darwin's theory – once a certain threshold of biology has been reached. But this hybrid solution is rather strange as it reveals a possible inconsistency. If we are faced with a mechanism, the KH suggests that we should select this above a fluctuation-based hypothesis. This is prescribed even when the KH is fully able to account for any level of organization compatible with the laws of physics. In the appendix to the paper, an informal calculation of the chance emergence of RNA polymers is provided, whose accuracy I can not judge within a few orders of magnitude. A very low probability is adduced and then used to support an infinite multiverse (O-ring) anthropic selection argument. But as I have stated above, an infinite model allows for infinite complexity at any level, and so the improbability of the polymer is not really crucial. On the basis of the KH are we really able to declare that any level of organization above replication and translation represents a sufficiently large fluctuation to be radically less probable, and therefore in need of natural selection? This is an interesting metaphysical idea, but not yet a scientific one.

So what's going on here? I think that we can identify two primary motivations for this paper: (1) a perhaps somewhat restricted application of scientific reasoning, and (2) an excessive dependence on natural selection.

I believe we have reached a stage of knowledge where we need to be pluralistic about the "scientific method". Most of science has the following features: (1)testable or refutable, (2) minimal, (3) extendable or generalizable, and (4) probable. Not all theories in science possess all of these properties, but all of them come up in debate in one form or another. The first or refutability is the Popperian gold standard. It defines the demarcation criterion of science from pseudoscience, distinguishing assertions that can garner consensus from matters of taste and prejudice. Theories that are not refutable in principle do not belong in science. The second is minimality and is closely related to formalization in mathematics. Science seeks the shortest description length for regularities in data. We can always overfit data and this leads to poor prediction out of sample. Science values minimality both because it aids comprehension and because it aids prediction. Good scientific theories are generalizable – Newtonian mechanics works equally well for apples and for planetary masses. Newton described this propertry as self-conformable. Darwins theory has the same property, it works for bacteria and blue whales. The probable criterion relates to inter-theoretic reduction or compatibility. Darwin made extensive use of artificial selection to explain natural selection. Darwin was building a prior in the space of concepts, in order to make natural selection intelligible. Theories that have no prior strike us as somewhat unconvincing.

We can now ask how well the KH performs by comparing it against the template of constituents of the loosely specified scientific method. The hypothesis fails the refutation test prima facie. The KH declares that all organizational levels are possible. Even if we were to discover a selective theory or a physical self organizing theory for replication and translation, the KH could still be true. Nothing can render it false. Presumably Koonin would no longer support the anthropic theory, not because it is any less possible, but because it lacks many of the ingredients that we have come to value in our scientific ideas. With regard to minimality, the theory is certainly minimal as it requires only the laws of physics, but here minimality does not serve the goal of prediction. Moreover, the theory is not at all extendable as every realization arises through a unique series of events. And we are not provided with a calculation for the probability of translation spontaneously emerging and fixing. Hence the theory does not fit well into what we normally think of as science.

It would seem that the primary motivation for the KH is the absence of a compelling Darwinian account for translation. But the Darwinian theory is only one of many alternative theories that seek to explain ordered states in nature. Before natural selection there existed physics, chemistry and geology. There is a growing interest in the possible role that abiotic dynamics have played in the origin of primary metabolism and primordial replicating systems. Indeed some of my colleagues have recently proposed that: "the reverse citric acid cycle is statistically favored among competing redox relaxation pathways under early-earth conditions and that this feature drove its emergence and also accounts for its evolutionary robustness and universality. The ability to enhance the rate of core reactions creates an energetic basis for selection of subsequent layers of biological complexity." (Smith and Morowitz 2004). This is an alternative to a selective account as under pre-enzymatic conditions, all reactions that relax the free energy of more stable, small inorganic molecules are impeded. Thus the essential constituents of primary metabolism emerge as a reduction sequence in a physical process of free energy minimization. This is a theory that requires a great deal of further research, both theoretical and empirical, but it conforms to the more traditional standards that we have come to require of a scientific approach.

Science seeks to understand the probable, and moreover assign some measure to this concept. There are many ideas that are possible-yet-untestable, but I see no reason to opt for these approaches when much traditional science remains to be done on the early origin of life, some of which suggests that life is far more probable than we have supposed. In an infinite multiverse all life is inevitable, and so we would have little reason to favor one parsimonious theory over another.

**Author's response**: *I agree with Krakauer that (as long as one is interested in a definition of science) a pluralistic definition would be required. However, I do not think this is the place to discuss such a definition in general terms. Suffice it to say that Popperian falsifiability of KH is strongly emphasized in this paper. The falsifiability of the general framework of KC is a more touchy matter as almost always is the case with general concepts, However, a full success of the search for an overarching selective principle that would precede and override replication of genetic material, might falsify KC in full. The work of Smith and Morowitz (, which is mentioned by Krakauer, (Smith, E., Morowitz, H.J*. **Universality in intermediary metabolism**. Proc Natl Acad Sci U S A. 2004;101:13168–73) *belongs in this area as well as even more detailed proposals of Pross (e.g., Pross, A*. **The driving force for life's emergence: kinetic and thermodynamic considerations**. J Theor Biol. 2003; 220:393–406). *Certainly, this is a legitimate and, potentially, interesting research direction. However, I agree with Krakauer that a lot of work is required to take these studies to a stage where it can be positively claimed that selection, in a meaningful sense, occurred before the advent of replication of digital carriers of genetic information. When and if that happens, KC could become obsolete in its entirety. Until then, however, I believe that a lot of caution is due. Indeed, without implying any accusation, I would note that there seems to be a rather slippery slope here: a non-critical insistence on selection prior to replication might come suspiciously close to some sort of "animism" (sensu Monod)*.

The history of science, is by one reading, interpreted as a catalogue of conceptual bottlenecks overcome by synthesizing unlikely concepts. The characteristics of a bottleneck are: an inability to make progress based on contemporary theory and data, a general impetus to search for radically new ideas, and a correlated tendency to default to extra-scientific modes of explanation based on putative forces and miracles. The general theory of relativity, quantum mechanics and natural selection all solved hard problems by combining hitherto unrelated concepts – non-Euclidean geometry and gravity, probability and mechanics, density regulation with environmental selection. Interestingly, all three theories have their non-scientific resonances, "everything is relative", "everything is subjective", and "everything was created" descending from the bottleneck period. The origin of life will require just such juxtapositions and has been generating just such non-scientific propositions.

**Author's response**:* I further enthusiastically agree with Krakauer that a productive study of the origins of life requires juxtaposition of "hitherto unrelated concepts". Indeed, this paper is an attempt, however imperfect one, to do just that. I do not believe, however, that the comparison of this work to the proverbially ridiculous "everything is relative" serves any purpose*.

## Reviewer 3: Sergei Maslov (Brookhaven National Laboratory)

Any manuscript invoking the anthropic selection principle to explain anything is bound to raise controversy. Let alone if what is being explained is the origin of Life itself.

I personally liked the style and the clear language of the manuscript. I also appreciated an honest back of the envelope estimate of the probability of primitive replication+translation machinery arising by pure chance: <10^(-1000) (sic!). At the very least this estimate provides a glimpse at the vast gap between the chance appearance of a single functional ribozyme replicase (plausible if its length is <100 nucleotides) and that of more complex entities such as a rudimentary machinery necessary for translation.

Author also clearly indicates how his theory could be falsified or modified by future discoveries. For example, the discovery of independently evolved life in our cosmic neighborhood would obviously put an end to any anthropic selection-based models of the origin of Life. On the other hand, a discovery of an evolutionary plausible path to the appearance of translational machinery in a full-fledged RNA world might simply reduce the severity of the anthropic selection needed to explain the origin of Life without completely eliminating the need for it.

The manuscript also postpones the discussion of plausibility of a "reactor" which uses raw materials to generate polynucleotides in sufficient quantity/density. It is simply stated that while "such "reactors" are not known", "networks of inorganic compartments existing at hydrothermal vents might be plausible candidates". It might turn out that the problem of lack or rarity of such reactors might dwarf the problem of low likelihood of the appearance of replication+translation machinery in one such reactor.

**Author response**: *It is curiously serendipitous that, while this manuscript was under review, new data have been published indicating that, given that monomers (nucleotides) are synthesized at any appreciable rate, the emergence of a "reactor" producing polymers (RNA molecules) in the vicinity of a hydrothermal vent, is not unlikely. These new findings are quoted in the revised paper (Refs. 56, 57)*.

While personally I hope we would find a way to explain the origin of biocomplexity without invoking the anthropic selection (perhaps, by some yet unknown mechanism of self-organization), one cannot deny that at the present state of affairs in understanding the origin of Life the anthropic selection at the very least provides a viable alternative.

**Author response**: *Actually, I think a rather common misunderstanding is involved here. I am convinced that the anthropic principle is unavoidable as part and parcel of **any **scenario for the origin of life, whether or not some still unknown principles of self-organization exist (they very well might). Plenty of anthropic selection is required to account for the formation galaxies and earth-like planets, prebiological organic syntheses etc etc. The real question is not whether or not anthropic selection is important (to me, there is no doubt whatever) but where is the transition between it and biological evolution, the threshold of complexity where Darwinian selection becomes possible (see *Fig. 1* in this paper). In this regard, one certainly may "hope" that the threshold is (considerably?) below the level of complexity associated with a coupled system of translation and replication (again, see *Fig. 1*) but so far there is no strong evidence or even a compelling model of biological evolution occurring at this stage*.

## Reviewer 4: Itai Yanai (Harvard University)

In this work, Eugene Koonin estimates the probability of arriving at a system capable of undergoing Darwinian evolution and comes to a cosmologically small number. With such an improbable event at hand, Koonin turns to a cosmological perspective in order to grasp its feasibility. He cites recent work in cosmology that highlights the vastness of the universe, where any series of events is necessarily played out an infinite number of times. This so-called "many-worlds in one" model essentially reconceives any chance event as a necessary one, where its (absolute) abundance is proportional to its chance of occurring.

The context of this article is framed by the current lack of a complete and plausible scenario for the origin of life. Koonin specifically addresses the front-runner model, that of the RNA-world, where self-replicating RNA molecules precede a translation system. He notes that in addition to the difficulties involved in achieving such a system is the paradox of attaining a translation system through Darwinian selection. That this is indeed a bona-fide paradox is appreciated by the fact that, without a shortage effort, a plausible scenario for translation evolution has not been proposed to date. There have been other models for the origin of life, including the ground-breaking Lipid-world model advanced by Segrè, Lancet and colleagues (reviewed in EMBO Reports (2000), 1(3), 217–222), but despite much ingenuity and effort, it is fair to say that all origin of life models suffer from astoundingly low probabilities of actually occurring.

Koonin's main contributions in this manuscript are two-fold: 1. a description of a minimal "breakthrough system" capable of priming Darwinian evolution" and most importantly 2. relating the issue of overcoming probability barriers by defaulting to the anthropic principle, which is supported by advances in cosmology. Together these provide for a model of the origin of life where the "breakthrough system" appears by chance and is sufficient to prime Darwinian evolution. Koonin distinguishes between a strong and weak form of this model. In the strong form, the entire "breakthrough system" occurs entirely by chance. While in the weak form, a less complex system (and thus more cosmologically common) is found that is able to achieve Darwinian evolution. Should such a less complex system be discovered, the breakthrough system as Koonin describes it will have been falsified; however, even this less complex system is likely to be vanishingly rare and consequently also requires the anthropic principle to account for its occurrence on Earth. Of course, the model would also be falsified if the "many-worlds in one" model is itself falsified.

Overall, this is a bold manuscript that promises to deeply influence the stream of thought on the origin of life. To my knowledge the present model represents the first one to account for the origin of life by explicitly invoking the anthropic principle. Whereas the sufficiency of time has been questioned for evolving life, invoking the anthropic principle allows for an elegant – albeit science-fiction-like – way out of this chicken and egg problem.

From this perspective, future advances in the field of the origin of life may better estimate the ubiquity of life in the universe by attempting to break down the "breakthrough system" into less complex parts. In the very extreme, future work may show that starting from just a simple assembly of molecules, non-anthropic principles can account for each step along the rise to the threshold of Darwinian evolution. Based upon the new perspective afforded to us by Koonin this now appears unlikely.

**Author's response**: *I agree with most of the statements in this constructive comment. Once again, however, I should note that, the way I see this situation, it is impossible to shun anthropic reasoning completely, whatever the advances of future work. As Yanai puts it, "in the very extreme", one could dream of non-anthropically explaining the entire sequence of evolutionary steps from monomers to a RNA-protein world. However, in the preceding history, an anthropic component inevitably will remain*.

*Perhaps, to complete the discussion, a final comment on the anthropic principle/selection/reasoning is due here. In all four reviews of this work, regardless of the other opinions of the reviewers, there is a strong emphasis on the anthropic principle that, in my view, is somewhat misplaced. Surely, the anthropic principle is important. However, I believe that it is secondary to the actual model of the uni(multi)verse. Indeed, the infinite repetition of all permissible histories in the MWO cosmology makes anthropic selection a straightforward epiphenomenon of the model (see text and *Table 1*). I should add that I also find it to be more satisfying philosophically that the model is put ahead of a "principle". Should the model be falsified, the status of the principle will become uncertain, and of course, the entire concept developed here, if not refuted in its entirety, will require a drastic revision (as rightly emphasized by Yanai)*.

## Authors' contributions

EVK conceived of the model, performed the calculations involved, and wrote the manuscript.

## Appendix

### Probabilities of the emergence, by chance, of different versions of the breakthrough system in an O-region: a toy calculation of the upper bounds

General assumptions: an *O*-region contains 10^22 ^stars and every 10^th ^star has a habitable planet, hence 10^21 ^habitable planets (undoubtedly, a gross over-estimation because, in reality, most stars have no planets at all, let alone habitable ones). Each planet is the size of earth and has a 10 kilometer (10^6 ^cm) thick habitable layer; hence the volume of the habitable layer is 4/3π[*R*^3^-(*R-l*)^3^] ≈ 5 × 10^24 ^cm^3^, where *R *is the radius of the planet and *l *is the thickness of the habitable layer. RNA synthesis occurs in 1% of the volume of the habitable layer, i.e., a volume *V *≈ 5 × 10^22 ^cm^3 ^is available for RNA synthesis (undoubtedly, a gross over-estimation because, in reality, there would be very few "RNA-making reactors"). Let the concentration of nucleotides in volume *V *and the rate of the synthesis of RNA molecules of size *n *(a free parameter depending on the specific model of the breakthrough stage; hereinafter *n*-mer) be 1 molecule/cm^3^/second (a gross overestimate for any sizable molecule; furthermore, the inverse dependence on *n*, which is expected to be strong, is disregarded). The time available after the Big Bang of the given *O*-region (as an upper bound) of all planets in it is 10^10 ^years ≈ 3 × 10^17 ^seconds. Then, the number of unique*n*-mers "tried out" during the time after the Big Bang is:

*S *≈ 5 × 10^22 ^× 10^21 ^× 3 × 10^17 ^≈ 1.5 × 10^61^.

Let us assume that, for the onset of biological evolution, a unique *n*-mer is required. The number of such sequences is *N *= 4^n ^≈10^0.6n^.

Then, the expectation of the number of times a unique *n*-mer emerges in an *O*-region is: *E *= *S*/*N *= 1.5 × 10^61^/10^0.6n ^and *n *= log(*E *× 1.5 × 10^61^)/0.6.

Substituting *E *= 1, we get *n *≈102 (nucleotides). Note that, because *n *is proportional to log*S*, the estimate is highly robust to the assumptions on the values of the contributing variables; e.g., a order of magnitude change in *S *will result in an increase or decrease of *n *by less than 2 nucleotides.

A ribozyme replicase consisting of ~100 nucleotides is conceivable, so, in principle, spontaneous origin of such an entity in a finite universe consisting of a single *O*-region cannot be ruled out in this toy model (again, the rate of RNA synthesis considered here is a deliberate, gross over-estimate).

The requirements for the emergence of a primitive, coupled replication-translation system, which is considered a candidate for the breakthrough stage in this paper, are much greater. At a minimum, spontaneous formation of:

- two rRNAs with a total size of at least 1000 nucleotides

- ~10 primitive adaptors of ~30 nucleotides each, in total, ~300 nucleotides

- at least one RNA encoding a replicase, ~500 nucleotides (low bound)is required. In the above notation, *n *= 1800, resulting in E <10^-1018^.

In other words, even in this toy model that assumes a deliberately inflated rate of RNA production, the probability that a coupled translation-replication emerges by chance in a single O-region is *P *< 10^-1018^. Obviously, this version of the breakthrough stage can be considered only in the context of a universe with an infinite (or, in the very least, extremely vast) number of *O*-regions.

The model considered here is not supposed to be realistic by any account. It only serves to illustrate the difference in the demands on chance for the origin of different versions of the breakthrough system (see Fig. 1) and hence the connections between these versions and different cosmological models of the universe.
